# Types and Aspects of Front-of-Package Labeling Preferred by Parents: Insights for Policy Making in China

**DOI:** 10.3390/nu14040800

**Published:** 2022-02-14

**Authors:** Jia Cui, Ruijie Yan, Thomas Astell-Burt, Enying Gong, Lutong Zheng, Xinxuan Li, Jingwen Zhang, Lin Xiang, Lihong Ye, Yiluan Hu, Yuxiang Tang, Chao Gao, Li Xiao, Yan Jiang, Ruitai Shao, Xiaoqi Feng, Juan Zhang, Yuexin Yang

**Affiliations:** 1 School of Population Medicine and Public Health, Peking Union Medical College, Chinese Academy of Medical Sciences, Beijing 100730, China; cj18800435072@163.com (J.C.); yanrj@sph.pumc.edu.cn (R.Y.); thomasab@uow.edu.au (T.A.-B.); gongenying@cams.cn (E.G.); zhang_jw0129@163.com (J.Z.); xianglinpumc@163.com (L.X.); yelihongpumc@163.com (L.Y.); huyiluan@126.com (Y.H.); tyxxkai@163.com (Y.T.); shaor@who.int (R.S.); 2 Population Wellbeing and Environment Research Lab (PowerLab), Faculty of Social Sciences, University of Wollongong, Wollongong, NSW 2522, Australia; xiaoqi.feng@unsw.edu.au; 3 School of Health and Society, Faculty of the Arts, Social Sciences, and Humanities, University of Wollongong, Wollongong, NSW 2522, Australia; 4 Menzies Centre for Health Policy, Sydney School of Public Health, University of Sydney, Sydney, NSW 2006, Australia; 5 School of Health Humanities, Peking University, Beijing 100191, China; zhenglt@pku.edu.cn (L.Z.); 1810120133@pku.edu.cn (X.L.); 6 Shijiazhuang Municipal Bureau of Statistics, Shijiazhuang 050011, China; 7 Key Laboratory of Trace Element Nutrition of National Health Commission, National Institute for Nutrition and Health, Chinese Center for Disease Control and Prevention, Beijing 100050, China; gaochao20090901@163.com; 8 Chinese Health Education Network, Chinese Center for Health Education, Beijing 100020, China; xiaoli19792022@163.com; 9 Chinese Nutrition Society, Beijing 100022, China; elisayanjiang@163.com; 10 School of Population Health, Faculty of Medicine and Health, University of New South Wales, Sydney, NSW 2052, Australia

**Keywords:** front-of-package labeling, MTL, Nutri-Score, warning labels, health logos: Smart Choice, GDA, preference

## Abstract

The WHO recommends front-of-package labeling (FOPL) to help parents make healthier food choices for their children. But which type of FOPL resonates with parents in China? We performed a cross-sectional study to investigate parental preferences for five widely used formats of FOPL. A multi-stage cluster sampling method was applied to selected parents of students in primary and secondary schools in six provinces and municipalities from July 2020 to March 2021. A close-ended questionnaire was used to collect demographic information, parents’ preferences for five FOPL in three dimensions, perceptions of the importance of nutrients labeled on FOPL, and prepackaged foods that need FOPL most. Chi-square tests were used to examine the characteristics among five groups. The results showed that multiple traffic lights (MTL) was preferred by parents, followed by warning labels. Parents thought the most needed nutrients to label were sugar, salt, and total fat. The top three prepackaged foods to label were “baked food”, “milk and dairy products” and “sugar-sweetened beverages”. Our findings indicate that nutrient-specific FOPL formats with interpretive aids were preferred by Chinese parents. These new findings can help inform the planning and implementation of FOPL in China and help Chinese parents make healthier food choices.

## 1. Introduction

Poor diet is largely responsible for worldwide non-communicable diseases (NCDs), causing 80% of deaths in China every year in particular [1]. According to Scientific Research Report on Chinese Dietary Guidelines (2021) [2], people in China are consuming excessive salt, edible oil, and added sugars. Prepackaged food and processed food are important components of those unhealthy diets and account for a growing proportion. China is now one of the world’s largest countries of prepackaged food consumption [3]. In particular, a comparative study from 12 countries reported that prepackaged food in China contains higher amounts of salt, fat, and sugar and is less healthier than other countries [1].

Making a healthier choice is very important in this severe food environment. To achieve this, the Chinese government has released national standards of the Nutrition Facts Panel (NFP) to regulate the labeling of required energy content, core nutrients, and the Nutrient Reference Value (NRV) of all pre-packaged foods [4]. However, the public’s understanding and usage of the NFP was poor, and many other countries have the same issues [5]. To address the problem, many countries have introduced front-of-package labeling (FOPL). FOPL is a simplified version of the NFP and a more effective form of nutrition labeling, allowing for a quick comparison among products by providing less information and a simpler expression form [6]. Most FOPL can be classified as reductive or interpretive. Reductive FOPL reduces the amount of information provided in the NFP but offers little interpretation. In contrast, interpretive FOPL provides judgement information on the specific nutrients, such as salt, sugar, and fat, or a summary indicator of the nutritional quality of a product. They may also use colors to indicate the healthiness of the food [7]. So far, over 50 countries have implemented FOPL of different types [8]; their data indicates that FOPL could help consumers to select healthier food effectively [9]. The WHO also recommended governments to implement FOPL system [6]. As a result, the Chinese government formulated a range of concrete measures including *“Promote the use of FOPL on food packaging”* in *“Healthy China Initiative 2019–2030”* [10].

In addition, FOPL has been considered an important strategy for ending childhood obesity [11]. According to the 2020 Report on Chinese Residents’ Chronic Diseases and Nutrition [12], the prevalence of overweight and obesity among children aged 6–17 was 19.0% [13]. China now has the highest number of obese children in the world [13]. Childhood obesity not only influences children’s present health and wellbeing, but also increases the risk for cardiovascular and metabolic diseases such as type 2 diabetes in adulthood [14,15]. Excessive consumption of unhealthy prepackaged foods, which contains excessive salt, fat and sugars is a risk factor for childhood obesity [16,17]. Parents are the main purchasers of children’s prepackaged foods. FOPL could be a very useful source of information to help parents make healthier decisions for their children easily. This need for an evidence-based approach is especially important for enabling parents to take pro-active action in helping reduce childhood obesity in China. But which FOPL is likely to resonate with Chinese parents?

Furthermore, WHO recommendations for FOPL system development should consider the difference in country context [6]. The New World Heart Federation (WHF) also stresses that consumer literacy rate and universal cultural norms on food and nutrients should be taken into account when implementing FOPL system [18]. To localize FOPL policies, many countries, such as the UK [19] and France [20], have made some efforts before promoting FOPL. A qualitative research published recently by our team has highlighted that China’s context differs from other countries [21]. There is an urgent need to identify a culturally and socioeconomically acceptable format of FOPL for Chinese consumers. Moreover, as there are many categories of pre-packaged food in China [22], it is not realistic to implement FOPL in all categories at once. Stakeholders from China recommend a pilot in the selective food categories before the implementation of FOPL nationwide [21].

This study aims to address the gap in knowledge by describing levels of preference for different FOPL with various characteristics among Chinese parents, as well as to identify their preferred nutrients and categories of prepackaged food. The findings will inform the planning and implementation of FOPL in China as a strategy for improving child health and reducing obesity.

## 2. Materials and Methods

### 2.1. Sample

A multi-stage cluster sampling method was applied to select parents of students registered in primary and secondary schools in six provinces and municipalities from July 2020 to March 2021. The sample size was calculated according to the formula $n={\left(\frac{\mu_{\alpha \;}}{\delta }\right)}^{2}P\left(1-P\right)$. Existing studies have shown that the awareness rates of nutrition labels of Chinese parents were from 45% to 86% [23,24,25]. Taking *P* = 45%, α=0.05, $\mu_{\alpha }=1.96$, and δ=0.1P, and considering a 20% non-response rate and 2 times expanded sample size for cluster sampling, the sample size was calculated to be 1174. According to the stratification of urban and rural areas, the total sample size was calculated as $\frac{1174}{\mathrm{layer}}\times 2\;\mathrm{layers}=2348$. We finally planned to select 2400 parents in total, and 400 parents for each province or municipality.

China can be generally divided into four major economic regions, including the eastern region, the central region, the western region, and the northeast region. First, we selected Beijing, Jiangsu, and Guangdong in the eastern region, Henan in the central region, Sichuan in the western region, and Heilongjiang in the northeast region. According to the level of economic development, the county-level administrative units of each province and municipality are divided into two levels, namely, urban areas (referring to county-level cities, and districts) and rural areas (referring to counties). Second, in the selected six provinces and municipalities, the provincial project team designated one county-level administrative unit for the urban areas and one for the rural areas. Since there are no rural areas in Beijing, a district with low economic circumstance in the urban areas was selected to replace it. A total of six county-level administrative units of the urban areas and 6 of the rural areas were selected finally. Third, for each selected county-level administrative unit, three regular primary schools and three regular secondary schools were selected [26]. A total of 72 schools were selected. In general, there are approximately 40–60 students in each class of primary schools or secondary schools in China. Fourth, to meet the requirement of sample size, one class in each school was selected; a total of 72 classes were selected. We investigated one of the parents of each student in the selected classes. Further details about the flow chart for sampling can be found in Figure S1.

The inclusion criteria were: (i) Parents of fifth-grade students at primary school or first-grade students at secondary school; and (ii) Parents with informed consent.

A total of 2548 parents participated in the study, and 141 parents without matched information of the students’ grade of school and snack habits were excluded in the analysis; thus, a total of 2407 parents were included in the analysis.

### 2.2. Data Collection

A close-ended online questionnaire adapted from previous studies [1,22,27,28,29,30,31] was administered to collect demographic information of parents and students, parents’ preferences for five formats of FOPL in three dimensions [27,28,29,30], parents’ perception of the importance of nutrients to be on FOPL [1,31], and prepackaged food that most needs to be on FOPL [22]. To test the rationality (i.e., usage of text, option settings, and clear expression) and feasibility (i.e., time spent and personnel allocation) of the questionnaire, a pilot survey was conducted among a convenience sample of ten adult residents and four students in grade five in elementary school. Based on the responses of the participants in the pilot, the questionnaire added a brief annotation of FOPL for better understanding.

The teachers were responsible for delivering the online questionnaire via Wenjuanxing (China’s platform to design electronic questionnaires) through class WeChat (Chinese social software, similar to WhatsApp and Snapchat) groups, and calling upon the parents to fill it out. The students of the classes without WeChat groups took the paper questionnaire home, let their parents fill out the questionnaire, and took it back to school on the second school day.

### 2.3. Definition and Evaluation of Preference for FOPL in Different Dimensions

#### 2.3.1. Parents’ Preference for Five Formats of FOPL

In this survey, the respondents were presented with 3 dimensions assessing their preference for the five formats of FOPL. The dimensions were as follows:

1 Which format of FOPL helps you select healthier food quickly?
2 Which format of FOPL attracts you most?
3 Which format of FOPL provides the information you need most?

**Figure 1:**
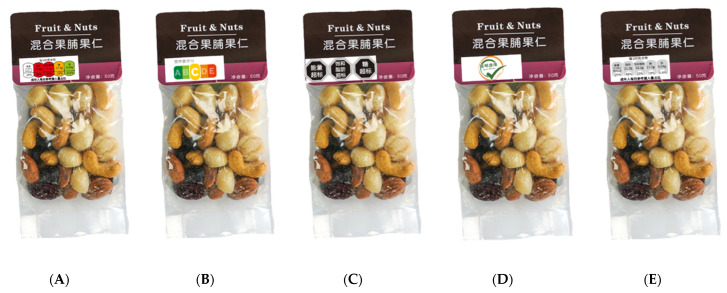
Five FOPL formats included in survey [28]: (**A**) MTL, provides nutrition information as guidancefor a set of nutrients, represented by the UK; (**B**) Nutri-Score, with five categories, using color coding and letters (from **A**–**E**) to summarize the healthfulness of products, represented by France; (**C**) warning labels, denoting foods that are high in certain critical nutrients, represented by Chile; (**D**) health logos: Smart Choice combines several criteria to establish one indication of the healthiness of a product and shows judgement or recommendation with no specific information; first FOP introduced by the Chinese Nutrition Society in China [32] (**E**) GDA, shows information only, with no specific judgement or recommendation; the GDA was replaced with warning labels in Mexico in 2021.

The five formats of FOPL were as follows (Figure 1) [27,28]:

- Interpretative nutrient-specific systems: multiple traffic lights (MTL)
- Interpretative summary indicator systems: Nutri-Score
- Interpretative nutrient-specific systems: warning labels
- Interpretative summary indicator systems: endorsement logos (Health logos: Smart Choice)
- Non-interpretive system: Guideline Daily Amount (GDA) system

#### 2.3.2. Parents’ Perception of the Importance of Nutrients on FOPL

The respondents were asked to answer the following question: ‘How important is it to you if the following nutrients are labeled on FOPL?’. The nutrients included energy, total fat, saturated fat (acid), trans fat (acid), carbohydrate, sugar and salt (sodium). The items were assessed on a 5-point Likert scale (very unimportant; unimportant; not sure; important; very important).

#### 2.3.3. Parents’ Perception of the Prepackaged Food Needing FOPL Most

The respondents were asked to answer the following question: ‘Which of the following prepackaged foods do you think need to be labeled FOPL most? (Multiple-choice question)’. The categories of prepackaged food were as follows (Figure S2 showed the sample pictures of these prepackaged foods) [22]:

- Chocolate, candy, etc. (chocolate bars, fondants, jelly, etc.)
- Sugar-sweetened beverages (Cola, fruit juice, lactic acid beverage, tea drinks, coconut juice, syrup of almond, etc.)
- Potato chips/crisps, and crispy rice
- Baked food (bread, pastries, moon cakes, biscuits, etc.)
- Seasoning sauces (ketchup, bean paste, salad dressing, barbecue sauce, etc.)
- Condiments (soy sauce, oyster sauce, etc.)
- Processed meat products (ham sausage, jerky, canned meat, bacon, etc.)
- Preserved food (pickles, preserved meat or fish, etc.)
- Convenience food (instant rice, instant noodles, quick-frozen food, self-heating hot pots, etc.)
- Milk and dairy products (pure milk, yogurt, cheese, etc., excluding milk drinks)
- Nuts, seeds, and dried fruits (peanuts, seeds, walnuts, dried fruits, etc.)

### 2.4. Assessment of Covariates

Covariates included parents and students’ basic characteristics. Parents’ data included age, residence, family roles, the highest level of educational attainment and number of children. Students’ data included grade of school, weight status perceived by parents, weight status defined by body mass index (BMI), dietary habits perceived by parents and snack habits.

### 2.5. Ethical Consideration

All respondents gave their informed consent for inclusion before they participated in the study. The study protocol was approved by the Chinese Center for Disease Control and Prevention Institutional Review Board (202025).

### 2.6. Statistical Analysis

Data preprocessing and database establishment were completed by SAS University edition software (Copyright © 2022, SAS Institute Inc., Cary, NC, USA), and statistical analysis was performed in IBM SPSS (version 25, SPSS Inc., Chicago, IL, USA). Categorical variables were presented as frequency and percent, and independent chi-square tests were used to examine the differences in the proportions of preference for five formats of FOPL between groups by subject characteristics. Post hoc comparisons were performed with a partition of the *χ*^2^ method for multiple comparisons. Continuous variables of abnormal distribution such as parents’ age were presented as medians and interquartiles, and the Kruskal–Wallis test was performed to compare the distribution among five groups by a preference for FOPL formats. A *p* value of < 0.05 (two-sided) was considered statistically significant.

## 3. Results

### 3.1. Characteristics of Chinese Parents Participating in the Study

The demographic characteristics are shown in Table 1. The median age of 2407 parents was 39.0 years (interquartile range, 36.0–43.0), 60.4% (1454/2407) were from urban areas, and 69.6% (1676/2407) were mothers. In addition, 42.8% of the parents had a high school/diploma degree, only 18.4% had a bachelor’s degree or above, and 49.0% of the families had two children. The mean age of the children was 13.2 (2.5) years, and nearly half of the children were studying in primary school. About 63.2% of parents thought their children’ weight status was normal, and 19.9% thought their children were very fat, but in fact, 21.2% of children were overweight or obese when defined by self-reported weight and height. A total of 48.4% of parents thought their children’s dietary practices were average, and 37.1% of children liked snacks or liked snacks very much.

### 3.2. FOPL Format Attracting Chinese Parents Most

Table 2 shows FOPL preference of attraction dimensions by demographic characteristics. Overall, MTL was the preferred FOPL, with 35.1% of the respondents nominating it as attracting them most. This was followed by warning labels at 21.9%, Smart Choice at 16.6%, GDA at 13.8%, and Nutri-Score at 12.5%.

Parents from rural areas were more likely to prefer MTL (42.4% vs. 30.4%, *p* < 0.001) compared with urban parents. Urban parents were more likely to indicate warning labels (26.6% vs. 14.8%, *p* < 0.001) compared with rural parents.

The difference in family roles was that fathers were more likely to think Nutri-Score attracted them most compared to mothers (14.9% vs. 11.5%, *p* = 0.018). Mothers were more likely to indicate Smart Choice compared to fathers (17.7% vs. 14.0%, *p* = 0.022).

There was a difference by education level; parents with a bachelor’s degree or higher educational achievement were significantly more likely to prefer warning labels (compared with middle school or below: 28.1% vs. 19.0%; compared with high school/diploma degree: 28.1% vs. 22.0%, all *p* < 0.05) and less likely to prefer Smart Choice (compared with middle school or below: 9.7% vs. 19.7%; compared with high school/diploma degree: 9.7% vs. 16.7%, all *p* < 0.05).

There was a difference by number of children in the family, the preference for warning labels being significantly higher among parents with one child (compared with families with two children: 26.1% vs. 19.7%; compared families with three or more children: 26.1% vs. 14.9%, all *p* < 0.05).

The difference in the grade of school was that the parents of secondary school students were more likely to think Nutri-Score attracted them most compared with those of primary school students (13.8% vs. 11.1%, *p* = 0.041).

Parents who thought their child had bad dietary habits were more likely to prefer MTL (39.2% vs. 31.7; 39.2% vs. 32.0%, all *p* < 0.05), and less likely to prefer warning labels (18.7% vs. 23.1%; 18.7% vs. 24.7, all *p* < 0.05).

Parents whose children liked snacks were more likely to prefer Smart Choice (19.9% vs. 14.7%, *p* = 0.001) and less likely to prefer GDA (11.1% vs. 15.6%, *p* = 0.010) compared with those whose children were neutral in liking snacks. 

There was no significant difference in parents’ FOPL preferences by children’s weight status perceived by parents or defined by BMI.

### 3.3. FOPL Format Providing Information Chinese Parents Need Most

MTL was still the preferred format with 42.4% of parents selecting it for “Which format provides the information you need most”. This was followed by GDA at 20.8% and the warning labels at 19.3%. A small proportion of the respondents preferred Smart Choice (9.8%) and Nutri-Score (7.8%).

The demographic characteristics of FOPL preference are shown in Table 3. Parents from rural areas were more likely to prefer MTL (52.5% vs. 35.8%, *p* < 0.00, *p* < 0.001) and Smart Choice (11.9% vs. 8.5%, *p* = 0.006) compared with urban parents. Urban parents were more likely to indicate warning labels (23.2% vs. 13.3%, *p* < 0.001) compared with rural parents.

There was a difference by education level. Bachelor’s degree and above parents were more likely to prefer MTL compared with middle school and below parents (47.7% vs. 39.7%, *p* = 0.006. Middle school and below parents were more likely to indicate Smart Choice (compared with high school/diploma degree: 12.9% vs. 8.8%; compared with bachelor’s degree and above: 12.9% vs. 5.4%, all *p* < 0.05) and GDA (compared with bachelor’s degree and above: 22.9% vs. 15.8%, *p* = 0.004).

Parents with one child were less likely to prefer Smart Choice compared with parents with two children (22.0% vs. 17.0%, *p* = 0.024).

Parents whose children liked snacks were more likely to prefer Smart Choice (compared with those whose children dislike snacks: 12.2% vs. 7.9%; compared with those whose children were neutral on liking snacks, 12.2% vs. 8.4%, all *p* < 0.05). 

There was no significant difference with parents’ preference for FOPL formats in family roles, children’s grade, dietary habits perceived by parents, and children’s weight status perceived by parents or defined by BMI.

### 3.4. FOPL Format Helping Chinese Parents Select Healthier Food Quickly Most

When it comes to “Which format helps you select healthier food quickly”, MTL was still the preferred FOPL, with 33.5% of the respondents selecting it. This was followed by warning labels at 24.2%, GDA at 17.7%, and Smart Choice at 15.5%. A small proportion of the respondents (9.5%) preferred Nutri-Score.

Table 4 shows the breakdown of FOPL preference according to demographic characteristic. Parents from rural areas were more likely to prefer MTL (40.5% vs. 29.0%, *p* < 0.001) and Smart Choice (17.4% vs. 13.5%, *p* = 0.009) compared with urban parents. Urban parents were more likely to indicate warning labels (28.1% vs. 18.2%, *p* < 0.001) compared with rural parents.

The one difference by gender was that fathers were more likely to indicate Nutri-Score compared to mothers (11.6% vs. 8.6%, *p* = 0.020).

Parents with bachelor’s degree and above education level were more likely to prefer warning labels compared with those with middle school and below education level (30.5% vs. 20.7%, *p* < 0.001). The bachelor’s degree and above parents were less likely to prefer Smart Choice (compared with middle school and below parents: 7.7% vs. 18.2%; compared with high school/diploma degree parents 7.7% vs. 15.5%, all *p* < 0.05).

The parents with one child were more likely to prefer warning labels (compared with the family with two children: 28.9% vs. 20.8%; compared with the family with three or more children: 28.9% vs. 20.7%, all *p* < 0.05).

Parents of primary school students were more likely to indicate MTL compared with secondary school children (36.3% vs. 30.9%, *p* = 0.004). While the secondary school parents were more likely to indicate Nutri-Score compared to primary school parents (10.7% vs. 8.3%, *p* = 0.044).

Parents who considered their children to have bad dietary habits were less likely to prefer warning labels compared with those whose children had average dietary habits (21.5% vs. 26.1%, *p* = 0.009).

Parents whose children liked snacks were more likely to identify Smart Choice (compared with those whose children dislike snacks: 18.0% vs. 9.2%; compared with those whose children neutral like snacks 18.0% vs. 13.8%, all *p* < 0.05) and less likely to identify GDA (compared with those whose children neutral like snacks: 15.1% vs. 19.3%, *p* = 0.010). 

There was no significant difference in parents’ preferences by children’s weight status perceived by parents or defined by BMI.

### 3.5. Preferences for FOPL by Residence Stratification

 Supplementary Materials Tables S1–S3 show the parental’ preference for five formats of FOPL by residence stratification. The results were consistent with the whole respondents. MTL was the preferred FOPL format in all three dimensions both on rural and urban levels. Fathers were more likely to indicate a preference for Nutri-Score compared to mothers. Parents with middle school or below were more likely to indicate Smart Choice, and those with bachelor’s degree or above were more likely to indicate warning labels. The family with one child were more likely to prefer warning labels. Parents who considered their children as having average dietary habits were more likely to prefer warning labels. The parents whose children liked snacks were more likely to identify Smart Choice.

### 3.6. Preference for Nutrients on FOPL and Prepackaged Food Labeled by FOPL

**Figure 2:**
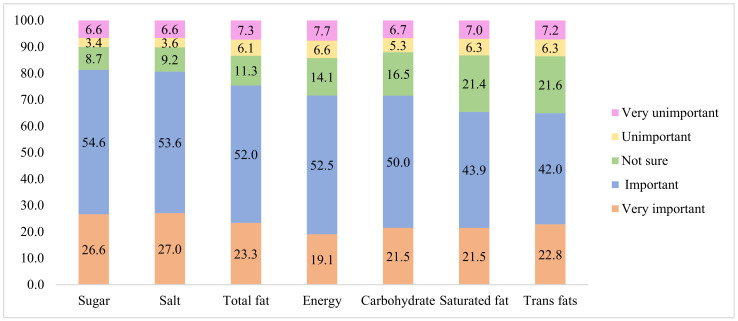
The nutrients preferred to be identified on FOPL (%).

**Figure 3:**
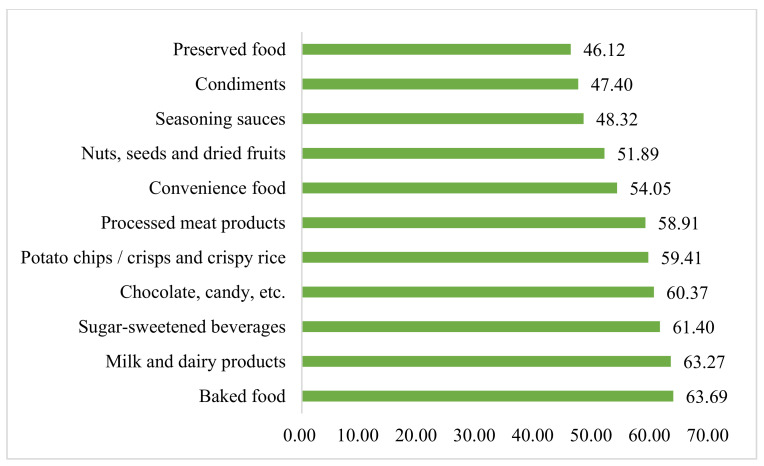
The preference to identify FOPL on 11 categories of prepackaged food (%).

The most preferred nutrient to be identified on FOPL was sugar at 81.3%. This was followed by salt (80.6%), total fat (75.4%), energy (71.6%), carbohydrates (71.5%), saturated fat (65.4%), and trans fats (64.9%) as showed in Figure 2. The top six categories of prepackaged food to identify FOPL preferred by parents were “baked food (63.7%)”, “milk and dairy products (63.3%)”, “sugar-sweetened beverages (61.4%)”, “chocolate, candy, etc. (60.3%)”, “potato chips/crisps, and crispy rice (59.4%)” and “processed meat products (58.9%)” as shown in Figure 3.

## 4. Discussion

This study provides important knowledge about the preference for FOPL among parents from a representative population from six provinces in China. Parents’ preferences were rated by multiple dimensions, including attraction, information provision, and helpfulness for making healthier choices. Among five potential FOPL choices, MTL was preferred by most participants as it matches parents’ needs and is likely to help them make healthier choices. In addition, Chinese parents preferred sugar, salt, and total fat to be identified on FOPL. They preferred “baked food”, “milk and dairy products”, and “sugar-sweetened beverages” labeled by FOPL. Such findings provide important implications for policy decision making.

Our study showed that the perception of FOPL was clustered according to consistent preferences for specific formats. MTL performed best, followed by warning labels, and Nutri-Score performed the worst. Previous work has demonstrated that consumers perceive that more information is better [33]. The present study reported parents were most in favor of MTL (42.4%), GDA (20.8%), and warning labels (19.3%) in providing information, and the interpretative summary indicator systems (Smart Choice and Nutri-Score) were insufficient. On the other hand, most consumers are not equipped to interpret all this information due to factors such as low levels of nutrition knowledge and time pressure [7]. A review showed that FOPL schemes incorporating text and symbolic color were easier to interpret than simply providing numeric information [34]. This is consistent with the present results showing that GDA was perceived to provide enough information yet is harder to use. In contrast, the results suggest that interpretive aids such as color were viewed favorably by parents, but an oversimplified format (Nutri-Score) risks excluding information that is desired by consumers and as a consequence being less desirable [7]. Previous studies suggested interpretative nutrient-specific systems (i.e., MTL and warning labels) providing information about specific nutrients rather than an overall indicator of healthiness had the most impact on knowledge [34,35]. This is consistent with the current finding that Nutri-Score was perceived as not providing enough information and being harder to interpret. This is an important message for policy makers: FOPL that provides information with interpretive aids, such as color, is viewed favorably by parents.

Our results indicate parents’ perceptions were relatively consistent across sociodemographic sub-groups, with few differences observed by parents’ family roles, the number of children in the family, students’ grade and weight status. The present study indicates that compared with parents residing in rural areas, urban-based parents were more likely to rate warning labels as providing enough information and as easier to use. Parents with higher education in both urban and rural areas were more likely to rate warning labels as providing enough information and as easier to use. It may be that parents with higher education are more accustomed to using more simplified nutrient-specific label such as warning labels. However, parents perceiving their children with poor dietary practices reported warning labels to provide the least information and as harder to use. Previous work reported that warning labels was least liked, but the easiest to interpret [7]. Furthermore, parents whose children like eating snacks reported the Smart Choice FOPL as a better provider of information and easier to use. This is consistent with previous findings that formats with interpretive elements showing judgements or recommendations are considered most useful [36].

It is worth noting that parents perceiving children with poor dietary practices may be less likely to interpret nutrient information and prefer interpretive formats with judgements. This is important for policy makers as interpretive formats of FOPL with judgements are easier to use and are favorably perceived by the parents whose children have unhealthy diets. The past five years has brought rapid innovation in labels that evaluate product unhealthfulness, which appear more effective in supporting consumers to choose nutritionally favorable products [37]. These include 10 nutrient-specific formats which used descriptive words (e.g., low, medium, high); meaningful colors (red, yellow, green, and black), and salient symbols (e.g., stop sign, traffic lights) to illustrate specific nutrient content [36]. Decision-makers developing strategies to introduce FOPL in China should consider using interpretive elements including subjective words, symbols, and color for consumers to use easily. Perceptions are just one dimension on which consumers’ reactions to FOPL can be assessed. Future work should consider how food choices are affected across different groups.

A review reported that 28 of 30 FOPL included one or more nutrients associated with increased NCD risk: sodium (28), saturated fat (22), total sugar (21), trans fat (8), total fat (8), and added sugar [36]. A significant body of evidence suggests that as part of a comprehensive approach to NCDs, nutrients and/or food components included in regulation should relate to evidence of diet-related risk. Packaged food and beverage products in China have been found to contain the highest levels of saturated fat and total sugar content, and is second only to India in terms of calorie content, according a study conducted across 12 countries and in over 390,000 food and beverage products [38]. In 2019, China announced its national health strategy, along with the ambitious goals of cutting dietary oil by 30–40%, salt by some 50%, and sugar by at least 17% nationwide by 2030 in tandem with the overarching “Healthy China 2030” initiative [39]. The present study also reports parents rated nutrients such as sugar, salt, and total fat most important for including in FOPL. Current WHO guidance indicates that they be focused on nutrients already required to be declared on back-of-package [36]. Labeling of salt and fat is mandatory for prepackaged food in China. Sugar is a new nutrient subject to mandatory declaration under the revised standard of nutrition labeling, GB28050. Hence, FOPL with nutrients of salt, sugar, and total fat along with interpretive elements may help guide the food industry to provide supplemental nutrition information on the front panel of packaging to facilitate consumers understanding’ and to help inform consumers’ decision-making that leads to healthier diets.

A qualitative study analyzing the context of FOPL implementation in China suggested that a pilot beginning the implementation of FOPL in selected food categories was recommended by stakeholders [21]. Our findings indicate that parents thought of “baked food”, “milk and dairy products”, and “sugar-sweetened beverages” as prepackaged foods needing FOPL. “Milk and dairy products” contain lots of nutrients, such as protein, amino acids, and vitamins [40,41]. Children need an adequate consumption of nutrients to maintain the body’s rapid growth and development. Regular use of “milk and dairy products” could be a reason why parents think it needs to be labeled. “backed food” and “sugar-sweetened beverage” are generally considered unhealthy as they contain excessive amounts of sugar, and fat, which often contribute to childhood obesity [16]. These prepackaged foods often make consumers feel confused about making a relatively healthier choice [19]. UK and France have similar considerations when introducing FOPL [19,20].

Our study was subject to some limitations. First, our study focused on the perception of FOPL labels and not on understanding or use of FOPL labels in purchasing situations. However, following the theoretical framework for the use of FOPL nutrition labels, favorable perception is a crucial pre-requisite for the effectiveness of a given label [42]. Second, the measures that were used in the study were not formally validated but based on scientific literature. They were derived from previously published work on perceptions of FOPL nutrition labeling. Finally, the use of an online survey may not perfectly recreate a real-world scenario in which participants interact with actual product labels. However, this type of survey is well suited for drawing attention to labeling attributes of interest.

## 5. Conclusions

Overall, MTL was the most preferred FOPL format among Chinese parents. Our study indicates a nutrient-specific (i.e., salt, sugar, and total fat) FOPL format with interpretive aids (i.e., color and judgement) may be viewed favorably by Chinese parents. Such a finding has implications for the introduction of FOPL in China.

## Figures and Tables

**Table 1:** Characteristics of Chinese parents participating in the study: *n*(%).

Characteristic	Total (*n* = 2407)	Urban (*n* = 1454)	Rural (*n* = 953)	*p* Value
***Parents***				
**Age**				0.679
*Mean* ± *SD*	39.0 (36.0, 43.0)	39.0 (37.0, 43.0)	39.0 (36.0, 43.0)	
**Family roles**				**0.016**
Father	731 (30.4)	415 (28.5)	316 (33.2)	
Mother	1676 (69.6)	1039 (71.5)	637 (66.8)	
**Highest level of educational attainment**				**<0.001**
Middle school and below	936 (38.9)	482 (33.1)	454 (47.6)	
High school/Diploma degree	1029 (42.8)	676 (46.5)	352 (37.0)	
Bachelor degree and above	442 (18.4)	296 (20.4)	146 (15.3)	
**Number of children in the family**				**<0.001**
1	1006 (41.8)	705 (48.5)	301 (31.6)	
2	1179 (49.0)	647 (44.5)	532 (55.8)	
≥3	222 (9.2)	102 (7.0)	120 (12.6)	
***Students***				
**Grade of school**				0.998
Primary school	1172 (48.7)	708 (48.7)	464 (48.7)	
Secondary school	1235 (51.3)	746 (51.3)	489 (51.3)	
**Weight status perceived by parents**				0.129
Very slim	376 (15.6)	243 (16.7)	133 (14.0)	
Normal	1522 (63.2)	903 (62.1)	619 (65.0)	
Very fat	478 (19.9)	297 (20.4)	181 (19.0)	
Not sure	31 (1.3)	11 (0.8)	20 (2.1)	
**Weight status defined by BMI**				0.660
Normal or below	1896 (78.8)	1141 (78.5)	755 (79.2)	
Overweight or obese	511 (21.2)	313 (21.5)	198 (20.8)	
**Dietary habits perceived by parents**				0.740
Very good/good	186 (7.7)	112 (7.7)	74 (7.8)	
Average	1166 (48.4)	710 (48.8)	456 (47.8)	
Not good/very bad	1055 (43.8)	632 (43.5)	423 (44.4)	
**Snack habits**				0.078
Dislike	152 (6.3)	93 (6.4)	59 (6.2)	
Neutral	1362 (56.6)	845 (58.1)	517 (54.2)	
Like or like very much	893 (37.1)	516 (35.5)	377 (39.6)	

**Table 2:** FOPL format attracting Chinese parents most: *n* (%).

Characteristics	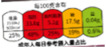	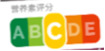	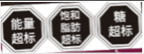	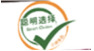	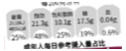	*p* Value
	MTL	Nutri-Score	Warning Labels	Smart Choice	GDA	
**Total**	846 (35.1)	301 (12.5)	528 (21.9)	399 (16.6)	333 (13.8)	
***Parents***						
**Age**						0.065
*Mean* ± *SD*	39.0 (36.0, 43.0)	40.0 (37.0, 44.0)	39.0 (36.0, 42.0)	39.0 (36.0, 43.0)	39.0 (36.5, 43.0)	
**Residence**						**<0.001**
Urban	442 (30.4) ^A^	203 (14.0) ^A^	387 (26.6) ^A^	224 (15.4)	198 (13.6)	
Rural	404 (42.4) ^B^	98 (10.3) ^B^	141 (14.8) ^B^	175 (18.4)	135 (14.2)	
**Family roles**						**0.036**
Father	255 (34.9)	109 (14.9) ^A^	169 (23.1)	102 (14.0) ^A^	96 (13.1)	
Mother	591 (35.3)	192 (11.5) ^B^	359 (21.4)	297 (17.7) ^B^	237 (14.1)	
**Highest level of educational attainment**						**<0.001**
Middle school and below	329 (35.1)	117 (12.5)	178 (19.0) ^A^	184 (19.7) ^A^	128 (13.7)	
High school/Diploma degree	366 (35.6)	116 (11.3)	226 (22.0) ^A^	172 (16.7) ^A^	149 (14.5)	
Bachelor’s degree and above	151 (34.2)	68 (15.4)	124 (28.1) ^B^	43 (9.7) ^B^	56 (12.7)	
**Number of children in the family**						**<0.001**
1	345 (34.3)	119 (11.8)	263 (26.1) ^A^	147 (14.6)	132 (13.1)	
2	420 (35.6)	159 (13.5)	232 (19.7) ^B^	208 (17.6)	160 (13.6)	
≥3	81 (36.5)	23 (10.4)	33 (14.9) ^B^	44 (19.8)	41 (18.5)	
***Students***						
**Grade of school**						0.134
Primary school	424 (36.2)	130 (11.1) ^A^	271 (23.1)	195 (16.6)	152 (13.0)	
Secondary school	422 (34.2)	171 (13.8) ^B^	257 (20.8)	204 (16.5)	181 (14.7)	
**Weight status perceived by parents**						0.441
Very slim	132 (35.1)	37 (9.8)	87 (23.1)	61 (16.2)	59 (15.7)	
Normal	543 (35.7)	195 (12.8)	326 (21.4)	259 (17.0)	199 (13.1)	
Very fat	159 (33.3)	65 (13.6)	113 (23.6)	73 (15.3)	68 (14.2)	
Not sure	12 (38.7)	4 (12.9)	2 (6.5)	6 (19.4)	7 (22.6)	
**Weight status defined by BMI**						0.414
Normal or below	678 (35.8)	240 (12.7)	408 (21.5)	318 (16.8)	252 (13.3)	
Overweight or obese	168 (32.9)	61 (11.9)	120 (23.5)	81 (15.9)	81 (15.9)	
**Dietary habits perceived by parents**						**0.003**
Very good/good	59 (31.7) ^A,B^	25 (13.4)	43 (23.1) ^A,B^	30 (16.1)	29 (15.6)	
Average	373 (32.0) ^B^	143 (12.3)	288 (24.7) ^B^	211 (18.1)	151 (13.0)	
Not good/very bad	414 (39.2) ^A^	133 (12.6)	197 (18.7) ^A^	158 (15.0)	153 (14.5)	
**Snack habits**						**0.006**
Dislike	59 (38.8)	20 (13.2)	30 (19.7)	21 (13.8) ^A,B^	22 (14.5) ^A,B^	
Neutral	476 (34.9)	160 (11.7)	314 (23.1)	200 (14.7) ^B^	212 (15.6) ^B^	
Like or like very much	311 (34.8)	121 (13.5)	184 (20.6)	178 (19.9) ^A^	99 (11.1) ^A^	

Note: ^A,B^ Within demographic groups (e.g., residence), different superscripts indicate a significant difference (*p* < 0.05).

**Table 3:** FOPL format providing information Chinese parents needed most: *n* (%).

Characteristics	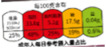	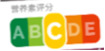	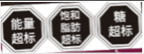	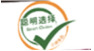	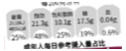	*p* Value
	MTL	Nutri-Score	Warning Labels	Smart Choice	GDA	
**Total**	1020 (42.4)	187 (7.8)	464 (19.3)	236 (9.8)	500 (20.8)	
***Parents***						
**Age**						0.054
*Mean* ± *SD*	40.0 (36.0, 43.0)	39.0 (38.0, 44.0)	39.0 (36.0, 42.0)	39.0 (36.0, 43.0)	39.0 (36.0, 43.0)	
**Residence**						**<0.001**
Urban	520 (35.8) ^A^	123 (8.5)	337 (23.2) ^A^	123 (8.5) ^A^	351 (24.1) ^A^	
Rural	500 (52.5) ^B^	64 (6.7)	127 (13.3) ^B^	113 (11.9) ^B^	149 (15.6) ^B^	
**Family roles**						0.366
Father	304 (41.6)	64 (8.8)	148 (20.2)	61 (8.3)	154 (21.1)	
Mother	716 (42.7)	123 (7.3)	316 (18.9)	175 (10.4)	346 (20.6)	
**Highest level of educational attainment**						**<0.001**
Middle school and below	372 (39.7) ^A^	65 (6.9)	164 (17.5)	121 (12.9) ^A^	214 (22.9) ^A^	
High school/Diploma degree	437 (42.5) ^A,B^	85 (8.3)	200 (19.4)	91 (8.8) ^B^	216 (21.0) ^A,B^	
Bachelor’s degree and above	211 (47.7) ^B^	37 (8.4)	100 (22.6)	24 (5.4) ^B^	70 (15.8) ^B^	
**Number of children in the family**						0.079
1	416 (41.4)	76 (7.6)	221 (22.0) ^A^	84 (8.3)	209 (20.8)	
2	509 (43.2)	99 (8.4)	201 (17.0) ^B^	124 (10.5)	246 (20.9)	
≥3	95 (42.8)	12 (5.4)	42 (18.9) ^A,B^	28 (12.6)	45 (20.3)	
***Students***						
**Grade of school**						0.218
Primary school	517 (44.1)	84 (7.2)	234 (20.0)	109 (9.3)	228 (19.5)	
Secondary school	503 (40.7)	103 (8.3)	230 (18.6)	127 (10.3)	272 (22.0)	
**Weight status perceived by parents**						0.225
Very slim	154 (41.0)	26 (6.9)	78 (20.7)	36 (9.6)	82 (21.8)	
Normal	664 (43.6)	109 (7.2)	281 (18.5)	159 (10.4)	309 (20.3)	
Very fat	190 (39.7)	49 (10.3)	102 (21.3)	36 (7.5)	101 (21.1)	
Not sure	12 (38.7)	3 (9.7)	3 (9.7)	5 (16.1)	8 (25.8)	
**Weight status defined by BMI**						0.280
Normal or below	821 (43.3)	141 (7.4)	356 (18.8)	190 (10.0)	388 (20.5)	
Overweight or obese	199 (38.9)	46 (9.0)	108 (21.1)	46 (9.0)	112 (21.9)	
**Dietary habits perceived by parents**						0.774
Very good/good	70 (37.6)	16 (8.6)	40 (21.5)	19 (10.2)	41 (22.0)	
Average	494 (42.4)	91 (7.8)	236 (20.2)	115 (9.9)	230 (19.7)	
Not good/very bad	456 (43.2)	80 (7.6)	188 (17.8)	102 (9.7)	229 (21.7)	
**Snack habits**						**0.008**
Dislike	67 (44.1)	11 (7.2)	23 (15.1)	12 (7.9) ^B^	39 (25.7)	
Neutral	556 (40.8)	107 (7.9)	286 (21.0)	115 (8.4) ^B^	298 (21.9)	
Like or like very much	397 (44.5)	69 (7.7)	155 (17.4)	109 (12.2) ^A^	163 (18.3)	

Note: ^A,B^ Within demographic groups (e.g., residence), different superscripts indicate a significant difference (*p* < 0.05).

**Table 4:** FOPL format helping Chinese parents select healthier food quickly most: *n* (%).

Characteristics	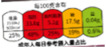	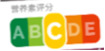	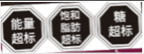	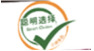	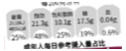	*p* Value
	MTL	Nutri-Score	Warning Labels	Smart Choice	GDA	
**Total**	807 (33.5)	229 (9.5)	582 (24.2)	363 (15.1)	426 (17.7)	
***Parents***						
**Age**						**<0.001**
*Mean* ± *SD*	39.0 (36.0, 43.0) ^A^	40.0 (38.0, 45.0) ^B^	39.0 (37.0, 42.0) ^B^	39.0 (36.0, 43.0) ^B^	39.0 (36.0, 43.0)^B^	
**Residence**						**<0.001**
Urban	421 (29.0) ^A^	152 (10.5)	409 (28.1) ^A^	197 (13.5) ^A^	275 (18.9)	
Rural	386 (40.5) ^B^	77 (8.1)	173 (18.2) ^B^	166 (17.4) ^B^	151 (15.8)	
**Family roles**						0.217
Father	236 (32.3)	85 (11.6) ^A^	172 (23.5)	107 (14.6)	131 (17.9)	
Mother	571 (34.1)	144 (8.6) ^B^	410 (24.5)	256 (15.3)	295 (17.6)	
**Highest level of educational attainment**						**<0.001**
Middle school and below	307 (32.8)	89 (9.5)	194 (20.7) ^A^	170 (18.2) ^A^	176 (18.8)	
High school/Diploma degree	342 (33.2)	89 (8.6)	253 (24.6) ^A,B^	159 (15.5) ^A^	186 (18.1)	
Bachelor’s degree and above	158 (35.7)	51 (11.5)	135 (30.5) ^B^	34 (7.7) ^B^	64 (14.5)	
**Number of children in the family**						**<0.001**
1	325 (32.3)	94 (9.3)	291 (28.9) ^A^	132 (13.1)	164 (16.3)	
2	404 (34.3)	123 (10.4)	245 (20.8) ^B^	193 (16.4)	214 (18.2)	
≥3	78 (35.1)	12 (5.4)	46 (20.7) ^B^	38 (17.1)	48 (21.6)	
***Students***						
**Grade of school**						**0.036**
Primary school	426 (36.3) ^A^	97 (8.3) ^A^	279 (23.8)	171 (14.6)	199 (17.0)	
Secondary school	381 (30.9) ^B^	132 (10.7) ^B^	303 (24.5)	192 (15.5)	227 (18.4)	
**Weight status perceived by parents**						0.307
Very slim	131 (34.8)	30 (8.0)	95 (25.3)	52 (13.8)	68 (18.1)	
Normal	518 (34.0)	144 (9.5)	345 (22.7)	242 (15.9)	273 (17.9)	
Very fat	149 (31.2)	50 (10.5)	136 (28.5)	62 (13.0)	81 (16.9)	
Not sure	9 (29.0)	5 (16.1)	6 (19.4)	7 (22.6)	4 (12.9)	
**Weight status defined by BMI**						0.820
Normal or below	636 (33.5)	180 (9.5)	452 (23.8)	294 (15.5)	334 (17.6)	
Overweight or obese	171 (33.5)	49 (9.6)	130 (25.4)	69 (13.5)	92 (18.0)	
**Dietary habits perceived by parents**						0.080
Very good/good	56 (30.1)	20 (10.8)	51 (27.4) ^A,B^	33 (17.7)	26 (14.0)	
Average	382 (32.8)	101 (8.7)	304 (26.1) ^B^	182 (15.6)	197 (16.9)	
Not good/very bad	369 (35.0)	108 (10.2)	227 (21.5) ^A^	148 (14.0)	203 (19.2)	
**Snack habits**						**0.005**
Dislike	49 (32.2)	21 (13.8)	40 (26.3)	14 (9.2) ^A^	28 (18.4) ^A,B^	
Neutral	448 (32.9)	120 (8.8)	343 (25.2)	188 (13.8) ^A^	263 (19.3) ^B^	
Like or like very much	310 (34.7)	88 (9.9)	199 (22.3)	161 (18.0) ^B^	135 (15.1) ^A^	

Note: ^A,B^ Within demographic groups (e.g., residence), different superscripts indicate a significant difference (*p* < 0.05).

## Data Availability

The data presented in this study are available on request from the corresponding author. The data are not publicly available according to private and confidential clauses stated in the informed consent.

## Supplementary Materials

The following supporting information can be downloaded at: https://www.mdpi.com/article/10.3390/nu14040800/s1, Figure S1: Flow chart for sampling, Figure S2: Eleven categories of prepackaged food included in this survey, Table S1: The FOPL format attracts parents most, stratified by residence: *n* (%), Table S2: The FOPL format provides information parents needed most, stratified by residence: *n* (%), Table S3: The FOPL format helps parents select healthier food quickly most, stratified by residence: *n* (%).
