# Supplementary material for: Types and Aspects of Front-of-Package Labeling Preferred by Parents: Insights for Policy Making in China
Source: Nutrients. 2022 Feb 14;14(4):800. doi: 10.3390/nu14040800 (PMC8878132; doi:10.3390/nu14040800)
Supplement: Supplementary file 1 [file nutrients-14-00800-s001.zip › nutrients-1563481-supplementary.pdf]

Supplementary Materials

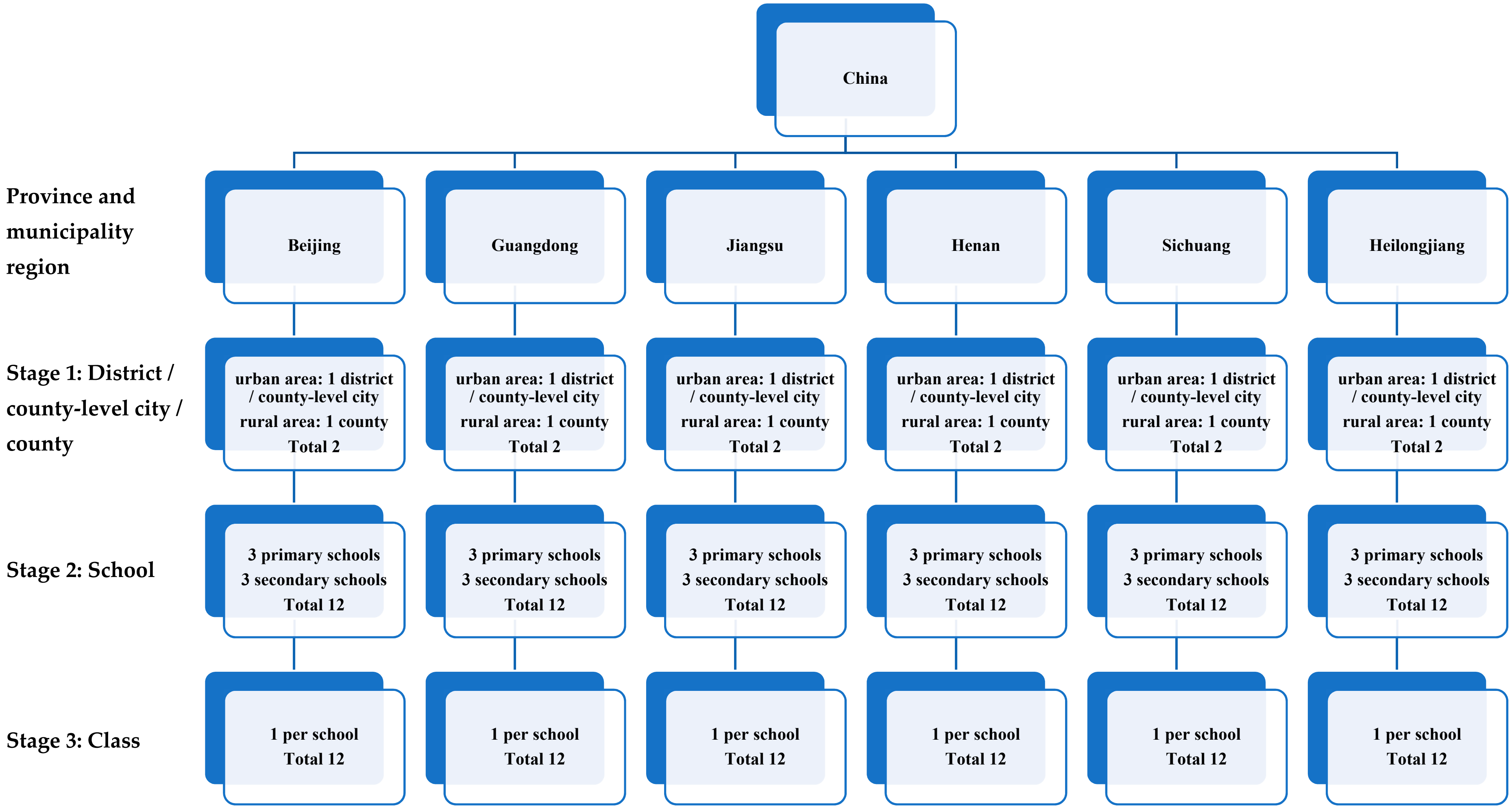

Figure S1. Flow chart for sampling.

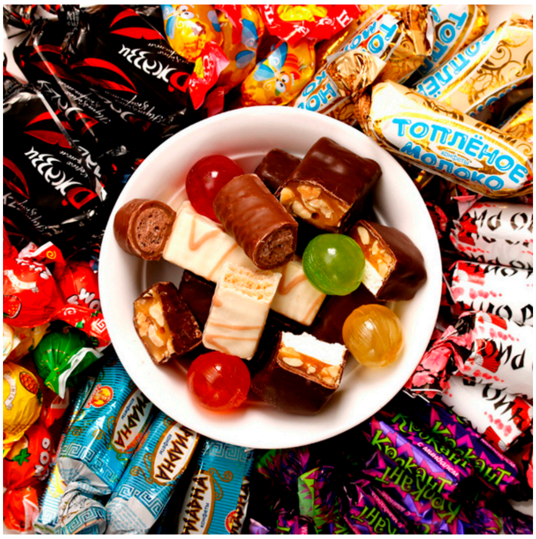

A. Chocolate, candy, etc.

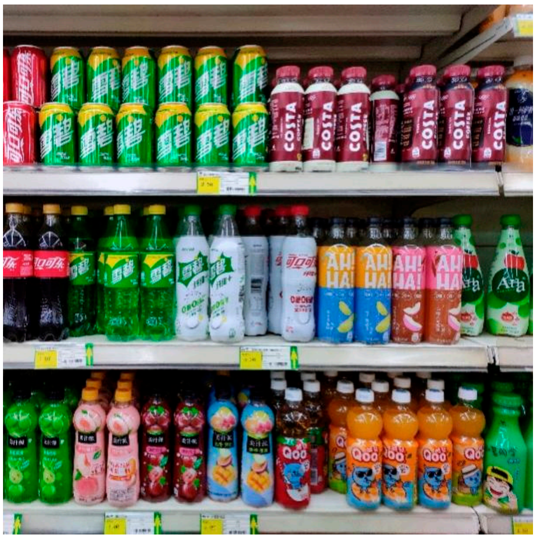

B. Sugar-sweetened beverages

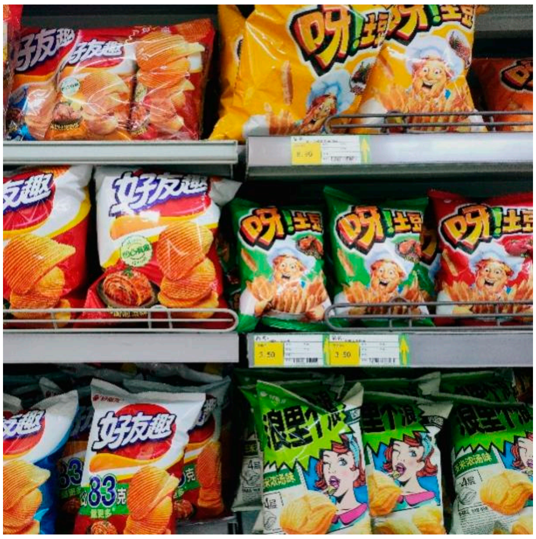

C. Potato chips / crisps, and crispy rice

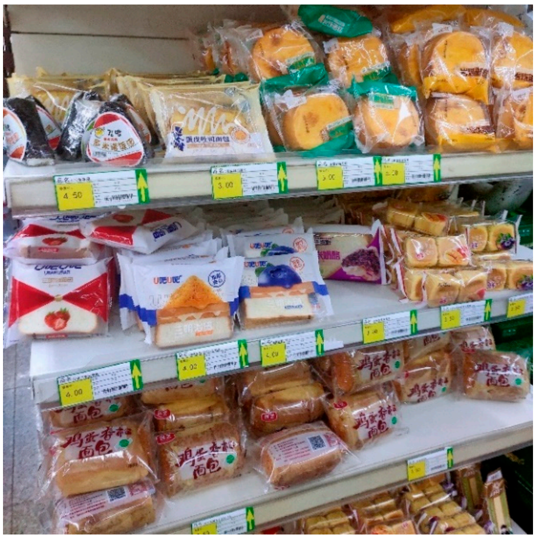

D. Baked food

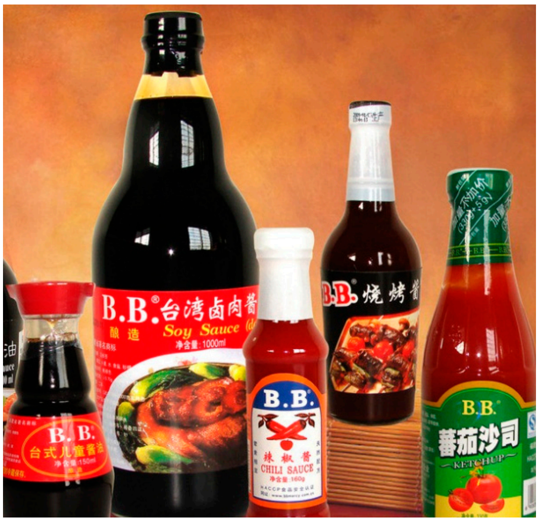

E. Seasoning sauces

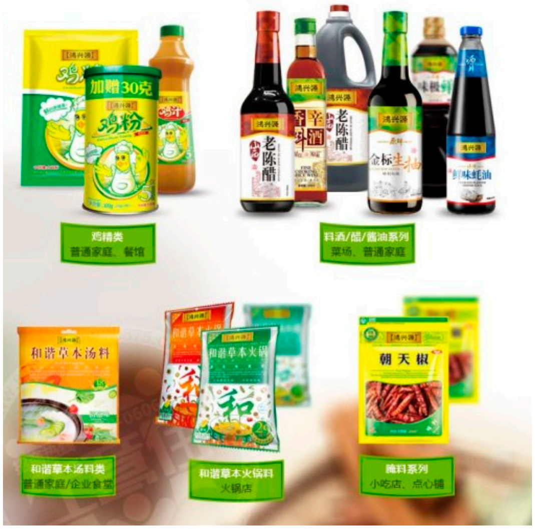

F. Condiments

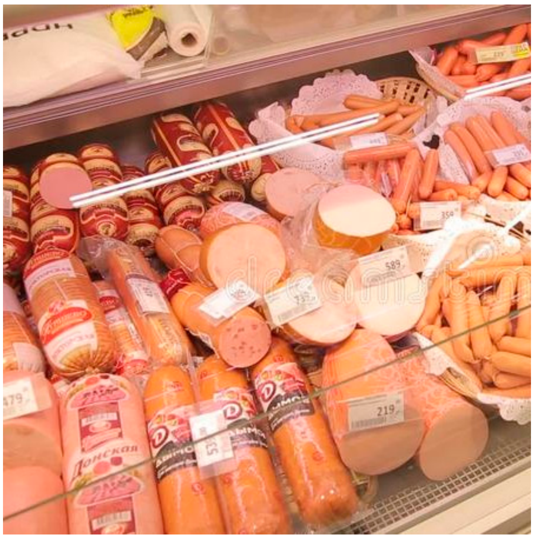

G. Processed meat products

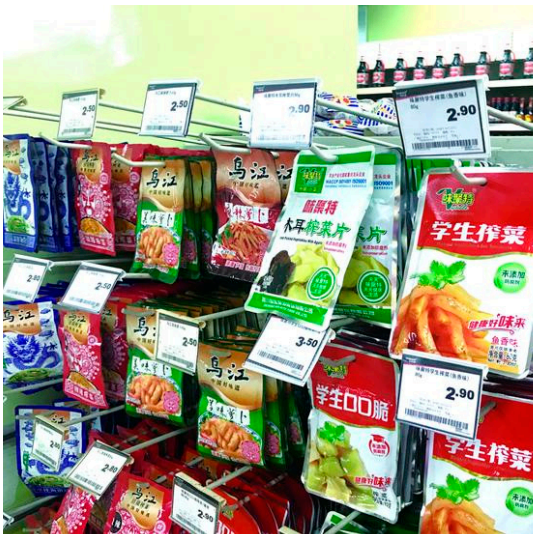

H. Preserved food

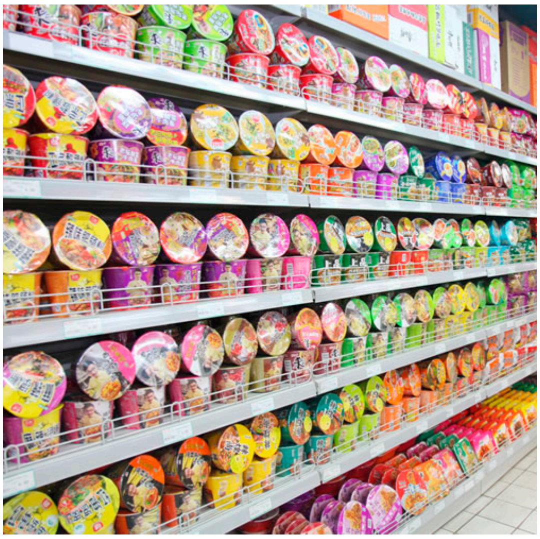

I. Convenience food

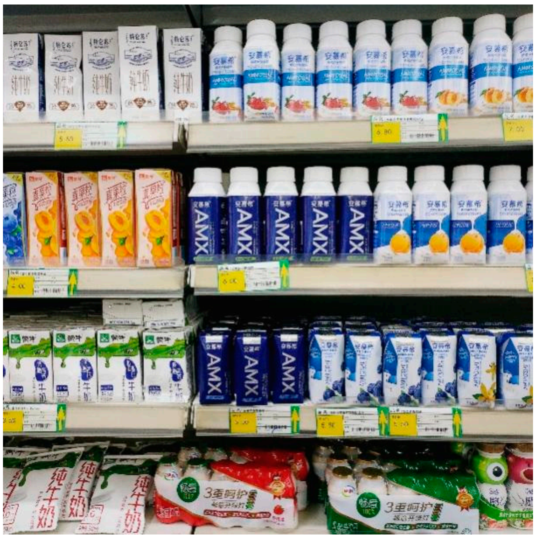

J. Milk and dairy products

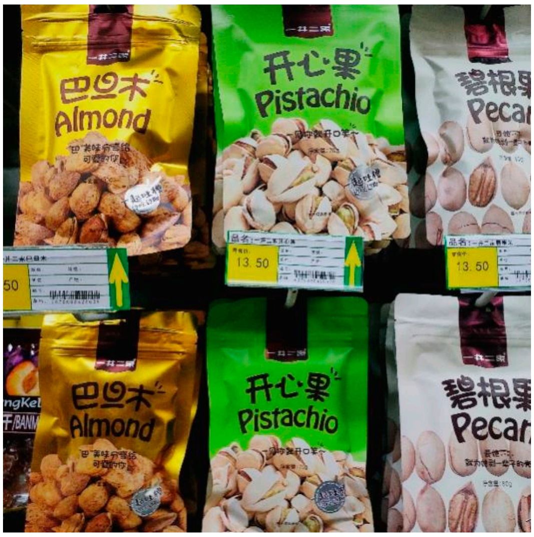

K. Nuts, seeds, and dried fruits

Figure S2. Eleven categories of prepackaged food included in this survey.

Table S1. The FOPL format attracts parents most, stratified by residence: n (%)

Characteristics	Total (n=2407)	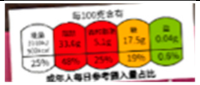			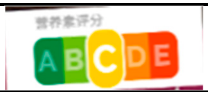			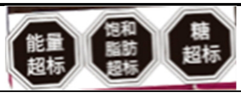			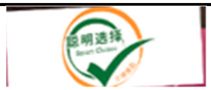			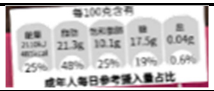			*P* value
		**MTL**			**Nutri-Score**			**Warning labels**			**Smart Choice**			**GDA**			
		Total	Urban	Rural	Total	Urban	Rural	Total	Urban	Rural	Total	Urban	Rural	Total	Urban	Rural	
		846 (35.1)	442 (30.4)	404 (42.4)	301 (12.5)	203 (14.0)	98 (10.3)	528 (21.9)	387 (26.6)	141 (14.8)	399 (16.6)	224 (15.4)	175 (18.4)	333 (13.8)	198 (13.6)	135 (14.2)	
Parents																	
**Age**																	**0.017**
Mean ± SD	39.9±5.2	40.0±5.2	40.4±5.1	39.5±5.3	40.7±5.3	40.4±5.1	41.5±5.7	39.5±4.8	39.1±4.6	40.8±5.3	39.8±5.5	40.0±5.6	39.5±5.4	39.6±5.3	39.2±5.2	40.1±5.5	
**Family roles**																	**0.036**
Father	731 (30.4)	255 (34.9)	134 (32.3)	121 (38.3)	109 (14.9)	66 (15.9)	43 (13.6)	169 (23.1)	118 (28.4)	51 (16.1)	102 (14.0)	53 (12.8)	49 (15.5)	96 (13.1)	44 (10.6)	52 (16.5)	
Mother	1676 (69.6)	591 (35.3)	308 (29.6)	283 (44.4)	192 (11.5)	137 (13.2)	55 (8.6)	359 (21.4)	269 (25.9)	90 (14.1)	297 (17.7)	171 (16.5)	126 (19.8)	237 (14.1)	154 (14.8)	83 (13.0)	
**Highest level of education attainment**																	**<0.001**
Middle school and below	936 (38.9)	329 (35.1)	133 (27.6)	196 (43.2)	117 (12.5)	72 (14.9)	45 (9.9)	178 (19.0)	120 (24.9)	58 (12.8)	184 (19.7)	94 (19.5)	90 (19.8)	128 (13.7)	63 (13.1)	65 (14.3)	
High school/Diploma degree	1029 (42.8)	366 (35.6)	213 (31.5)	153 (43.3)	116 (11.3)	86 (12.7)	30 (8.5)	226 (22.0)	177 (26.2)	49 (13.9)	172 (16.7)	101 (14.9)	71 (20.1)	149 (14.5)	99 (14.6)	50 (14.2)	
Bachelor degree and above	442 (18.4)	151 (34.2)	96 (32.4)	55 (37.7)	68 (15.4)	45 (15.2)	23 (15.8)	124 (28.1)	90 (30.4)	34 (23.3)	43 (9.7)	29 (9.8)	14 (9.6)	56 (12.7)	36 (12.2)	20 (13.7)	
**Number of child in the family**																	**<0.001**
1	1006 (41.8)	345 (34.3)	215 (30.5)	130 (43.2)	119 (11.8)	85 (12.1)	34 (11.3)	263 (26.1)	205 (29.1)	58 (19.3)	147 (14.6)	105 (14.9)	42 (14.0)	132 (13.1)	95 (13.5)	37 (12.3)	
2	1179 (49.0)	420 (35.6)	191 (29.5)	229 (43.0)	159 (13.5)	109(16.8)	50 (9.4)	232 (19.7)	161 (24.9)	71 (13.3)	208 (17.6)	101 (15.6)	107 (20.1)	160 (13.6)	85 (13.1)	75 (14.1)	
⩾3	222 (9.2)	81 (36.5)	36 (35.3)	45 (37.5)	23 (10.4)	9 (8.8)	14 (11.7)	33 (14.9)	21 (20.6)	12 (10.0)	44 (19.8)	18 (17.6)	26 (21.7)	41 (18.5)	18 (17.6)	23 (19.2)	
Students																	
**Grade of school**																	0.134
Primary school	1172 (48.7)	424 (36.2)	232 (32.8)	192 (41.4)	130 (11.1)	80 (11.3)	50 (10.8)	271 (23.1)	201 (28.4)	70 (15.1)	195 (16.6)	103 (14.5)	92 (19.8)	152 (13.0)	92 (13.0)	60 (12.9)	
Secondary school	1235 (51.3)	422 (34.2)	210 (28.2)	212 (43.4)	171 (13.8)	123 (16.5)	48 (9.8)	257 (20.8)	186 (24.9)	71 (14.5)	204 (16.5)	121 (16.2)	83 (17.0)	181 (14.7)	106 (14.2)	75 (15.3)	
**Weight status Perceived by parents**																	0.441
Very slim	376 (15.6)	132 (35.1)	78 (32.1)	54 (40.6)	37 (9.8)	25 (10.3)	12 (9.0)	87 (23.1)	64 (26.3)	23 (17.3)	61 (16.2)	43 (17.7)	18 (13.5)	59 (15.7)	33 (13.6)	26 (19.5)	
Normal	1522 (63.2)	543 (35.7)	271 (30.0)	272 (43.9)	195 (12.8)	128 (14.2)	67 (10.8)	326 (21.4)	247 (27.4)	79 (12.8)	259 (17.0)	136 (15.1)	123 (19.9)	199 (13.1)	121 (13.4)	78 (12.6)	
Very fat	478 (19.9)	159 (33.3)	92 (31.0)	67 (37.0)	65 (13.6)	48 (16.2)	17 (9.4)	113 (23.6)	74 (24.9)	39 (21.5)	73 (15.3)	42 (14.1)	31 (17.1)	68 (14.2)	41 (13.8)	27 (14.9)	
Not sure	31 (1.3)	12 (38.7)	1 (9.1)	11 (55.0)	4 (12.9)	2 (18.2)	2 (10.0)	2 (6.5)	2 (18.2)	0 (0.0)	6 (19.4)	3 (27.3)	3 (15.0)	7 (22.6)	3 (27.3)	4 (20.0)	
**Weight status defined by BMI**																	0.414
Normal or below	1896 (78.8)	678 (35.8)	340 (29.8)	338 (44.8)	240 (12.7)	157 (13.8)	83 (11.0)	408 (21.5)	313 (27.4)	95 (12.6)	318 (16.8)	178 (15.6)	140 (18.5)	252 (13.3)	153 (13.4)	99 (13.1)	
Overweight or obesity	511 (21.2)	168 (32.9)	102 (32.6)	66 (33.3)	61 (11.9)	46 (14.7)	15 (7.6)	120 (23.5)	74 (23.6)	46 (23.2)	81 (15.9)	46 (14.7)	35 (17.7)	81 (15.9)	45 (14.4)	36 (18.2)	
**Dietary habits perceived by parents**																	**0.003**
Very good/good	186 (7.7)	59 (31.7)	27 (24.1)	32 (43.2)	25 (13.4)	17 (15.2)	8 (10.8)	43 (23.1)	33 (29.5)	10 (13.5)	30 (16.1)	15 (13.4)	15 (20.3)	29 (15.6)	20 (17.9)	9 (12.2)	
Average	1166 (48.4)	373 (32.0)	194 (27.3)	179 (39.3)	143 (12.3)	97 (13.7)	46 (10.1)	288 (24.7)	216 (30.4)	72 (15.8)	211 (18.1)	113 (15.9)	98 (21.5)	151 (13.0)	90 (12.7)	61 (13.4)	
Not good/very bad	1055 (43.8)	414 (39.2)	221 (35.0)	193 (45.6)	133 (12.6)	89 (14.1)	44 (10.4)	197 (18.7)	138 (21.8)	59 (13.9)	158 (15.0)	96 (15.2)	62 (14.7)	153 (14.5)	88 (13.9)	65 (15.4)	
**Snacks habits**																	**0.006**
Dislike	152 (6.3)	59 (38.8)	33 (35.5)	26 (44.1)	20 (13.2)	11 (11.8)	9 (15.3)	30 (19.7)	20 (21.5)	10 (16.9)	21 (13.8)	13 (14.0)	8 (13.6)	22 (14.5)	16 (17.2)	6 (10.2)	
Neutral	1362 (56.6)	476 (34.9)	257 (30.4)	219 (42.4)	160 (11.7)	106 (12.5)	54 (10.4)	314 (23.1)	231 (27.3)	83 (16.1)	200 (14.7)	121 (14.3)	79 (15.3)	212 (15.6)	130 (15.4)	82 (15.9)	
Like or like very much	893 (37.1)	311 (34.8)	152 (29.5)	159 (42.2)	121 (13.5)	86 (16.7)	35 (9.3)	184 (20.6)	136 (26.4)	48 (12.7)	178 (19.9)	90 (17.4)	88 (23.3)	99 (11.1)	52 (10.1)	47 (12.5)	

Table S2. The FOPL format provides information parents needed most, stratified by residence: n (%)

Characteristics	Total (n=2407)	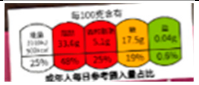 MTL			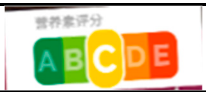 Nutri-Score			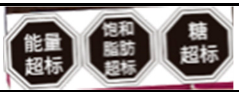 Warning labels			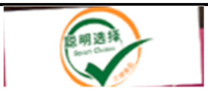 Smart Choice			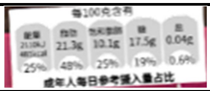 GDA			*P* value
		Total	Urban	Rural	Total	Urban	Rural	Total	Urban	Rural	Total	Urban	Rural	Total	Urban	Rural	
		1020 (42.4)	520 (35.8)	500 (52.5)	187 (7.8)	123 (8.5)	64 (6.7)	464 (19.3)	337 (23.2)	127 (13.3)	236 (9.8)	123 (8.5)	113 (11.9)	500 (20.8)	351 (24.1)	149 (15.6)	
Parents																	
**Age**																	**0.048**
Mean ± SD	39.9±5.2	40.2±5.4	40.5±5.1	39.9±5.6	40.4±4.5	40.6±4.1	40.0±5.2	39.6±5.1	39.2±4.9	40.5±5.4	39.8±5.4	40.0±5.5	39.5±5.2	39.5±5.2	39.1±5.3	40.4±4.9	
**Family roles**																	0.366
Father	731 (30.4)	304 (41.6)	144 (34.7)	160 (50.6)	64 (8.8)	45 (10.8)	19 (6.0)	148 (20.2)	101 (24.3)	47 (14.9)	61 (8.3)	28 (6.7)	33 (10.4)	154 (21.1)	97 (23.4)	57 (18.0)	
Mother	1676 (69.6)	716 (42.7)	376 (36.2)	340 (53.4)	123 (7.3)	78 (7.5)	45 (7.1)	316 (18.9)	236 (22.7)	80 (12.6)	175 (10.4)	95 (9.1)	80 (12.6)	346 (20.6)	254 (24.4)	92 (14.4)	
**Highest level of education attainment**																	**<0.001**
Middle school and below	936 (38.9)	372 (39.7)	142 (29.5)	230 (50.7)	65 (6.9)	31 (6.4)	34 (7.5)	164 (17.5)	110 (22.8)	54 (11.9)	121 (12.9)	59 (12.2)	62 (13.7)	214 (22.9)	140 (29.0)	74 (16.3)	
High school/Diploma degree	1029 (42.8)	437 (42.5)	244 (36.1)	193 (54.7)	85 (8.3)	68 (10.1)	17 (4.8)	200 (19.4)	155 (22.9)	45 (12.7)	91 (8.8)	48 (7.1)	43 (12.2)	216 (21.0)	161 (23.8)	55 (15.6)	
Bachelor degree and above	442 (18.4)	211 (47.7)	134 (45.3)	77 (52.7)	37 (8.4)	24 (8.1)	13 (8.9)	100 (22.6)	72 (24.3)	28 (19.2)	24 (5.4)	16 (5.4)	8 (5.5)	70 (15.8)	50 (16.9)	20 (13.7)	
**Number of child in the family**																	0.079
1	1006 (41.8)	416 (41.4)	261 (37.0)	155 (51.5)	76 (7.6)	55 (7.8)	21 (7.0)	221 (22.0)	169 (24.0)	52 (17.3)	84 (8.3)	57 (8.1)	27 (9.0)	209 (20.8)	163 (23.1)	46 (15.3)	
2	1179 (49.0)	509 (43.2)	223 (34.5)	286 (53.8)	99 (8.4)	62 (9.6)	37 (7.0)	201 (17.0)	143 (22.1)	58 (10.9)	124 (10.5)	56 (8.7)	68 (12.8)	246 (20.9)	163 (25.2)	83 (15.6)	
⩾3	222 (9.2)	95 (42.8)	36 (35.3)	59 (49.2)	12 (5.4)	6 (5.9)	6 (5.0)	42 (18.9)	25 (24.5)	17 (14.2)	28 (12.6)	10 (9.8)	18 (15.0)	45 (20.3)	25 (24.5)	20 (16.7)	
Students																	
**Grade of school**																	0.218
Primary school	1172 (48.7)	517 (44.1)	272 (38.4)	245 (52.8)	84 (7.2)	49 (6.9)	35 (7.5)	234 (20.0)	172 (24.3)	62 (13.4)	109 (9.3)	54 (7.6)	55 (11.9)	228 (19.5)	161 (22.7)	67 (14.4)	
Secondary school	1235 (51.3)	503 (40.7)	248 (33.2)	255 (52.1)	103 (8.3)	74 (9.9)	29 (5.9)	230 (18.6)	165 (22.1)	65 (13.3)	127 (10.3)	69 (9.2)	58 (11.9)	272 (22.0)	190 (25.5)	82 (16.8)	
**Weight status perceived by parents**																	0.225
Very slim	376 (15.6)	154 (41.0)	90 (37.0)	64 (48.1)	26 (6.9)	18 (7.4)	8 (6.0)	78 (20.7)	56 (23.0)	22 (16.5)	36 (9.6)	22 (9.1)	14 (10.5)	82 (21.8)	57 (23.5)	25 (18.8)	
Normal	1522 (63.2)	664 (43.6)	324 (35.9)	340 (54.9)	109 (7.2)	70 (7.8)	39 (6.3)	281 (18.5)	202 (22.4)	79 (12.8)	159 (10.4)	82 (9.1)	77 (12.4)	309 (20.3)	225 (24.9)	84 (13.6)	
Very fat	478 (19.9)	190 (39.7)	103 (34.7)	87 (48.1)	49 (10.3)	33 (11.1)	16 (8.8)	102 (21.3)	77 (25.9)	25 (13.8)	36 (7.5)	17 (5.7)	19 (10.5)	101 (21.1)	67 (22.6)	34 (18.8)	
Not sure	31 (1.3)	12 (38.7)	3 (27.3)	9 (45.0)	3 (9.7)	2 (18.2)	1 (5.0)	3 (9.7)	2 (18.2)	1 (5.0)	5 (16.1)	2 (18.2)	3 (15.0)	8 (25.8)	2 (18.2)	6 (30.0)	
**Weight status defined by BMI**																	0.280
Normal or below	1896 (78.8)	821 (43.3)	410 (35.9)	411 (54.4)	141 (7.4)	94 (8.2)	47 (6.2)	356 (18.8)	262 (23.0)	94 (12.5)	190 (10.0)	101 (8.9)	89 (11.8)	388 (20.5)	274 (24.0)	114 (15.1)	
Overweight or obesity	511 (21.2)	199 (38.9)	110 (35.1)	89 (44.9)	46 (9.0)	29 (9.3)	17 (8.6)	108 (21.1)	75 (24.0)	33 (16.7)	46 (9.0)	22 (7.0)	24 (12.1)	112 (21.9)	77 (24.6)	35 (17.7)	
**Dietary habits perceived by parents**																	0.774
Very good/good	186 (7.7)	70 (37.6)	34 (30.4)	36 (48.6)	16 (8.6)	11 (9.8)	5 (6.8)	40 (21.5)	30 (26.8)	10 (13.5)	19 (10.2)	10 (8.9)	9 (12.2)	41 (22.0)	27 (24.1)	14 (18.9)	
Average	1166 (48.4)	494 (42.4)	250 (35.2)	244 (53.5)	91 (7.8)	63 (8.9)	28 (6.1)	236 (20.2)	166 (23.4)	70 (15.4)	115 (9.9)	65 (9.2)	50 (11.0)	230 (19.7)	166 (23.4)	64 (14.0)	
Not good/very bad	1055 (43.8)	456 (43.2)	236 (37.3)	220 (52.0)	80 (7.6)	49 (7.8)	31 (7.3)	188 (17.8)	141 (22.3)	47 (11.1)	102 (9.7)	48 (7.6)	54 (12.8)	229 (21.7)	158 (25.0)	71 (16.8)	
**Snacks**																	**0.008**
Dislike	152 (6.3)	67 (44.1)	36 (38.7)	31 (52.5)	11 (7.2)	7 (7.5)	4 (6.8)	23 (15.1)	20 (21.5)	3 (5.1)	12 (7.9)	6 (6.5)	6 (10.2)	39 (25.7)	24 (25.8)	15 (25.4)	
Neutral	1362 (56.6)	556 (40.8)	290 (34.3)	266 (51.5)	107 (7.9)	70 (8.3)	37 (7.2)	286 (21.0)	200 (23.7)	86 (16.6)	115 (8.4)	67 (7.9)	48 (9.3)	298 (21.9)	218 (25.8)	80 (15.5)	
Like or like very much	893 (37.1)	397 (44.5)	194 (37.6)	203 (53.8)	69 (7.7)	46 (8.9)	23 (6.1)	155 (17.4)	117 (22.7)	38 (10.1)	109 (12.2)	50 (9.7)	59 (15.6)	163 (18.3)	109 (21.1)	54 (14.3)	

Table S3. The FOPL format helps parents select healthier food quickly most, stratified by residence: n (%)

Characteristics	Total (n=2407)	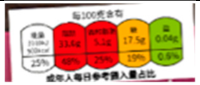 MTL			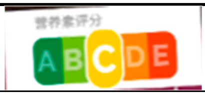 Nutri-Score			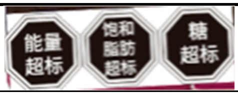 Warning labels			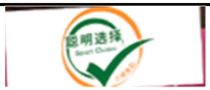 Smart Choice			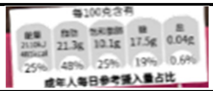 GDA			*P* value
		Total	Urban	Rural	Total	Urban	Rural	Total	Urban	Rural	Total	Urban	Rural	Total	Urban	Rural	
		807 (33.5)	421 (29.0)	386 (40.5)	229 (9.5)	152 (10.5)	77 (8.1)	582 (24.2)	409 (28.1)	173 (18.2)	363 (15.1)	197 (13.5)	166 (17.4)	426 (17.7)	275 (18.9)	151 (15.8)	
Parents																	
**Age**																	**<0.001**
Mean ± SD	39.9±5.2	39.6±5.2	39.9±5.0	39.4±5.4	41.3±5.1	41.2±4.8	41.5±5.7	39.7±5.0	39.4±5.0	40.4±5.0	39.9±5.3	39.9±5.3	39.8±5.4	39.9±5.4	39.5±5.3	40.6±5.5	
**Family roles**																	0.217
Father	731 (30.4)	236 (32.3)	112 (27.0)	124 (39.2)	85 (11.6)	55 (13.3)	30 (9.5)	172 (23.5)	114 (27.5)	58 (18.4)	107 (14.6)	51 (12.3)	56 (17.7)	131 (17.9)	83 (30.2)	48 (15.2)	
Mother	1676 (69.6)	571 (34.1)	309 (29.7)	262 (41.1)	144 (8.6)	97 (9.3)	47 (7.4)	410 (24.5)	295 (28.4)	115 (18.1)	256 (15.3)	146 (14.1)	110 (17.3)	295 (17.6)	192 (18.5)	103 (16.2)	
**Highest level of education attainment**																	**<0.001**
Middle school and below	936 (38.9)	307 (32.8)	115 (23.9)	192 (42.3)	89 (9.5)	44 (9.1)	45 (9.9)	194 (20.7)	135 (28.0)	59 (13.0)	170 (18.2)	90 (18.7)	80 (17.6)	176 (18.8)	98 (20.3)	78 (17.2)	
High school/Diploma degree	1029 (42.8)	342 (33.2)	197 (29.1)	145 (41.1)	89 (8.6)	77 (11.4)	12 (3.4)	253 (24.6)	182 (26.9)	71 (20.1)	159 (15.5)	86 (12.7)	73 (20.7)	186 (18.1)	134 (19.8)	52 (14.7)	
Bachelor degree and above	442 (18.4)	158 (35.7)	109 (36.8)	49 (33.6)	51 (11.5)	31 (10.5)	20 (13.7)	135 (30.5)	92 (31.1)	43 (29.5)	34 (7.7)	21 (7.1)	13 (8.9)	64 (14.5)	43 (14.5)	21 (14.4)	
**Number of child in the family**																	**<0.001**
1	1006 (41.8)	325 (32.3)	207 (29.4)	118 (39.2)	94 (9.3)	66 (9.4)	28 (9.3)	291 (28.9)	217 (53.1)	74 (24.6)	132 (13.1)	94 (13.3)	38 (12.6)	164 (16.3)	121 (17.2)	43 (14.3)	
2	1179 (49.0)	404 (34.3)	183 (28.3)	221 (41.5)	123 (10.4)	81 (12.5)	42 (7.9)	245 (20.8)	165 (25.5)	80 (15.0)	193 (16.4)	89 (13.8)	104 (19.5)	214 (18.2)	129 (19.9)	85 (16.0)	
⩾3	222 (9.2)	78 (35.1)	31 (30.4)	47 (39.2)	12 (5.4)	5 (4.9)	7 (5.8)	46 (20.7)	27 (26.5)	19 (15.8)	38 (17.1)	14 (13.7)	24 (20.0)	48 (21.6)	25 (24.5)	23(19.2)	
Students																	
**Grade of school**																	**0.036**
Primary school	1172 (48.7)	426 (36.3)	236 (33.3)	190 (40.9)	97 (8.3)	60 (8.5)	37 (8.0)	279 (23.8)	200 (28.2)	79 (17.0)	171 (14.6)	85 (12.0)	86 (18.5)	199 (17.0)	127 (17.9)	72 (15.5)	
Secondary school	1235 (51.3)	381 (30.9)	185 (24.8)	196 (40.1)	132 (10.7)	92 (12.3)	40 (8.2)	303 (24.5)	209 (28.0)	94 (19.2)	192 (15.5)	112 (15.0)	80 (16.4)	227 (18.4)	148 (19.8)	79 (16.2)	
**Weight status perceived by parents**																	0.307
Very slim	376 (15.6)	131 (34.8)	79 (32.5)	52 (39.1)	30 (8.0)	24 (9.9)	6 (4.5)	95 (25.3)	66 (27.2)	29 (21.8)	52 (13.8)	33 (13.6)	19 (14.3)	68 (18.1)	41 (16.9)	27 (20.3)	
Normal	1522 (63.2)	518 (34.0)	254 (28.1)	264 (42.6)	144 (9.5)	89 (9.9)	55 (8.9)	345 (22.7)	252 (27.9)	93 (15.0)	242 (15.9)	128 (14.2)	114 (18.4)	273 (17.9)	180 (19.9)	93 (15.0)	
Very fat	478 (19.9)	149 (31.2)	88 (29.6)	61 (33.7)	50 (10.5)	38 (12.8)	12 (6.6)	136 (28.5)	87 (29.3)	49 (27.1)	62 (13.0)	32 (10.8)	30 (16.6)	81 (16.9)	52 (17.5)	29 (16.0)	
Not sure	31 (1.3)	9 (29.0)	0 (0.0)	9 (45.0)	5 (16.1)	1 (9.1)	4 (20.0)	6 (19.4)	4 (36.4)	2 (10.0)	7 (22.6)	4 (36.4)	3 (15.0)	4 (12.9)	2 (18.2)	2 (10.0)	
**Weight status defined by BMI**																	0.820
Normal or below	1896 (78.8)	636 (33.5)	318 (27.9)	318 (42.1)	180 (9.5)	119 (10.4)	61 (8.1)	452 (23.8)	324 (28.4)	128 (17.0)	294 (15.5)	160 (14.0)	134 (17.7)	334 (17.6)	220 (19.3)	114 (15.1)	
Overweight or obesity	511 (21.2)	171 (33.5)	103 (32.9)	68 (34.3)	49 (9.6)	33 (10.5)	16 (8.1)	130 (25.4)	85 (27.2)	45 (22.7)	69 (13.5)	37 (11.8)	32 (16.2)	92 (18.0)	55 (17.6)	37 (18.7)	
**Dietary habits perceived by parents**																	**0.080**
Very good/good	186 (7.7)	56 (30.1)	27 (24.1)	29 (39.2)	20 (10.8)	14 (12.5)	6 (8.1)	51 (27.4)	37 (33.0)	14 (18.9)	33 (17.7)	18 (16.1)	15 (20.3)	26 (14.0)	16 (14.3)	10 (13.5)	
Average	1166 (48.4)	382 (32.8)	205 (28.9)	177 (38.8)	101 (8.7)	69 (9.7)	32 (7.0)	304 (26.1)	204 (28.7)	100 (21.9)	182 (15.6)	94 (13.2)	88 (19.3)	197 (16.9)	138 (19.4)	59 (12.9)	
Not good/very bad	1055 (43.8)	369 (35.0)	189 (29.9)	180 (42.6)	108 (10.2)	69 (10.9)	39 (9.2)	227 (21.5)	168 (26.6)	59 (13.9)	148 (14.0)	85 (13.4)	63 (14.9)	203 (19.2)	121 (19.1)	82 (19.4)	
**Eat snack**																	**0.005**
Dislike	152 (6.3)	49 (32.2)	28 (30.1)	21 (35.6)	21 (13.8)	12 (12.9)	9 (15.3)	40 (26.3)	30 (32.3)	10 (16.9)	14 (9.2)	5 (5.4)	9 (15.3)	28 (18.4)	18 (19.4)	10 (16.9)	
Neutral	1362 (56.6)	448 (32.9)	241(28.5)	207 (40.0)	120 (8.8)	80 (9.5)	40 (7.7)	343 (25.2)	236 (27.9)	107 (20.7)	188 (13.8)	110 (13.0)	78 (15.1)	263 (19.3)	178 (21.1)	85 (16.4)	
Like or like very much	893 (37.1)	310 (34.7)	152 (29.5)	158 (41.9)	88 (9.9)	60 (11.6)	28 (7.4)	199 (22.3)	143 (27.7)	56 (32.4)	161 (18.0)	82 (15.9)	79 (21.0)	135 (15.1)	79 (15.3)	56 (14.9)	
